# Supplementary material for: Biotransformation of p-xylene into terephthalic acid by engineered Escherichia coli
Source: Nat Commun. 2017 May 31;8:15689. doi: 10.1038/ncomms15689 (PMC5460024; doi:10.1038/ncomms15689)
Supplement: Supplementary Information — Supplementary Figures, Supplementary Tables and Supplementary References [file ncomms15689-s1.pdf]

## SUPPLEMENTARY FIGURES

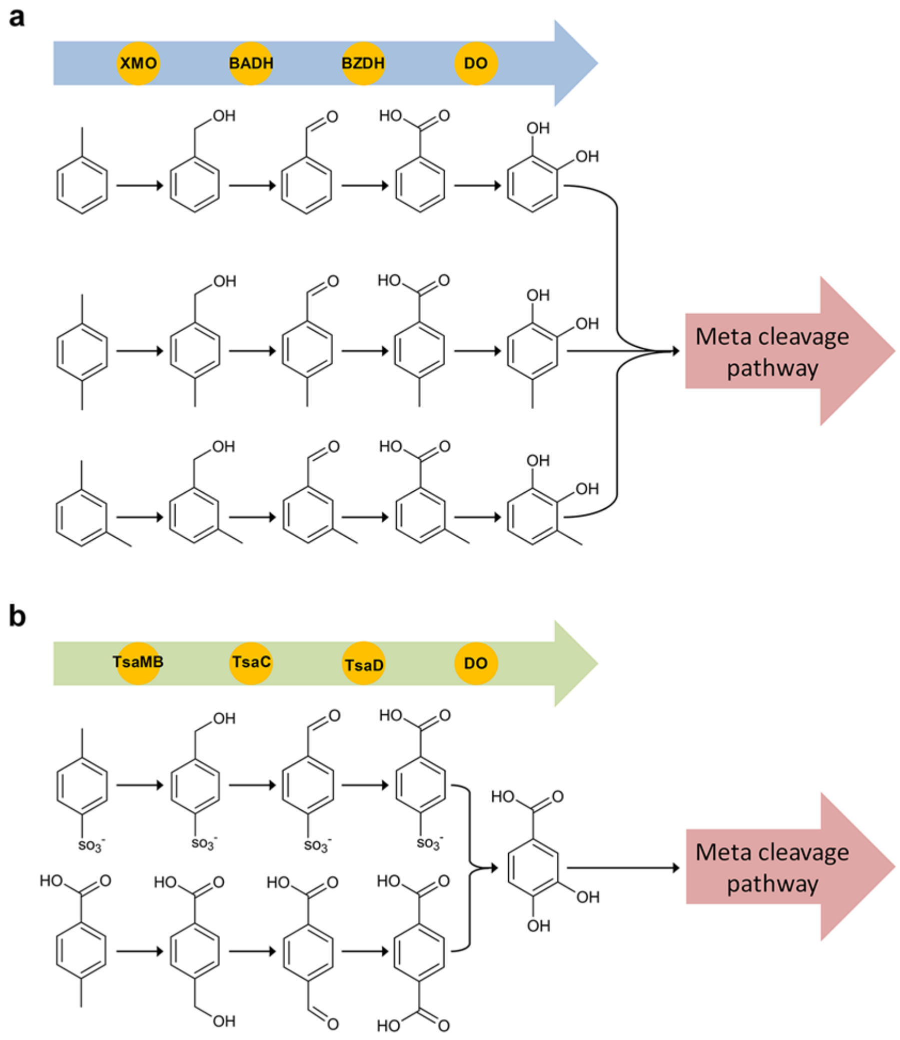


**Supplementary Figure 1. Natural degradation pathways of toluene and xylenes including *p*X and *m*X (a), *p*TS and *p*TA (b) in *P. putida* and *C. testosteroni* T-2, respectively.** Enzymes: XMO, xylene monooxygenase; BADH, benzyl alcohol dehydrogenase; BZDH, benzaldehyde dehydrogenase; DO, dioxygenase; *p*TS, *p*-toluene sulfonate; TsaMB, *p*-toluene sulfonate monooxygenase; TsaC, 4-carboxybenzyl alcohol dehydrogenase; TsaD, 4-carboxybenzaldehyde dehydrogenase.

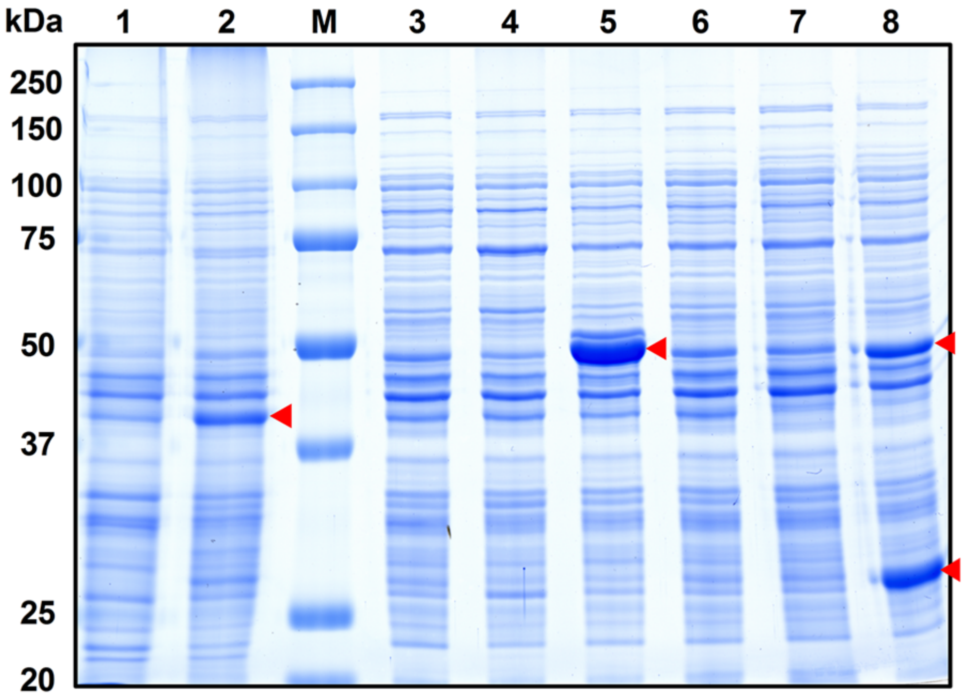


**Supplementary Figure 2. SDS-PAGE analysis of the overexpression of heterologous pathway enzymes.** Lane M, protein marker; lane 1, membrane fraction of DH5α harboring pTrc99A; lane 2, membrane fraction of DH5α harboring pTrcMA (XylM arrow-pointed); lane 3, soluble fraction of DH5α harboring pTrc99A; lane 4, soluble fraction of DH5α harboring pTrcMA; lane 5, soluble fraction of DH5α harboring pTrcC (XylC arrow-pointed); lane 6, soluble fraction of DH5α harboring pTrcMB; lane 7, soluble fraction of DH5α harboring pTac15K; lane 8, soluble fraction of DH5α harboring pTacCD (TsaC lower arrow-pointed, TsaD upper arrow-pointed). The expected molecular masses of XylM (membrane-bound), XylA, XylC, TsaM, TsaA, TsaC and TsaD are 43.2, 38.4, 52.9, 39.7, 34.3, 26.6 and 51.1 kDa, respectively.

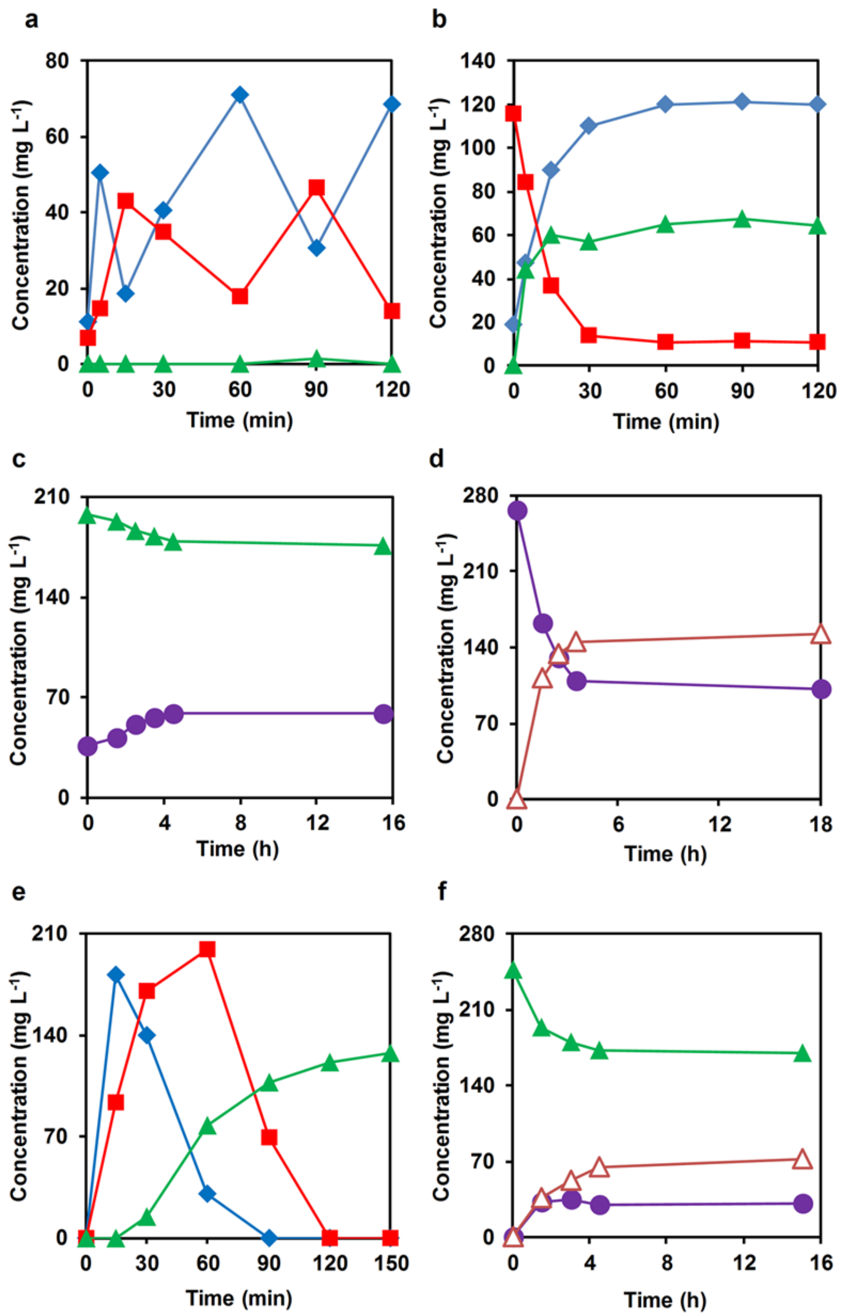


**Supplementary Figure 3. Characterization of each steps of conversion by whole-cell activity assay against recombinant *E. coli* cells.** Recombinant cells employed are *E. coli* DH5α harboring (**a**) pTrcMA, (**b**) pTrcC, (**c**) pTrcMB, (**d**) pTacCD, (**e**) pTrcMAC, and (**f**) pTacMBCD in the reaction mixtures containing substrates *p*X, *p*TALD, *p*TA, 4-CBAL, *p*X and *p*TA, respectively. The concentrations of substrates added in the reaction mixture are specified in

Methods section, while the substrate concentrations shown in above figures are actually measured values. It should be noted in (**b**) that the initial substrate (*p*TALD) concentration (118 mg $L^{-1}$) is much lower than that (200 mg $L^{-1}$) added in the reaction mixture. This is due to the time delay in measuring the concentrations in carrying out multiple reactions, while the conversion reaction of *p*TALD to *p*TALC is quite fast. The *p*X consumption profile is not shown owing to its insolubility in aqueous solution. Data for the wild-type strain as a control are not shown. Symbols are: blue diamond, *p*TALC; red rectangle, *p*TALD; solid green triangle, *p*TA; purple circle, 4-CBAL; open brown triangle, TPA.

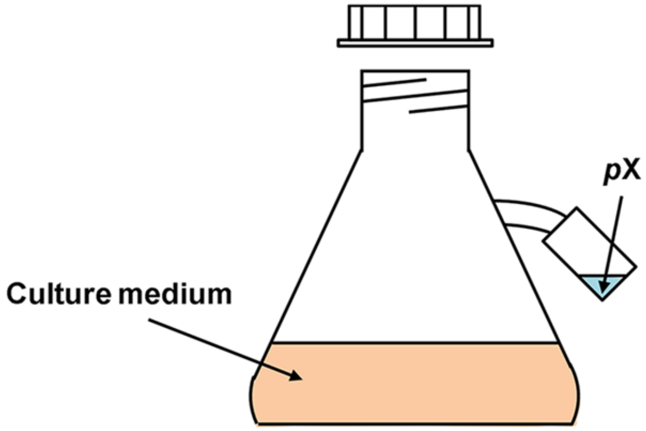


**Supplementary Figure 4. Schematic for the designed flask cultivation setup for *p*X to TPA conversion.** The screw-cap flask (250 mL) containing 50 mL of culture medium is connected with a small attached vial into which *p*X is loaded and comes into contact with culture medium by evaporation.

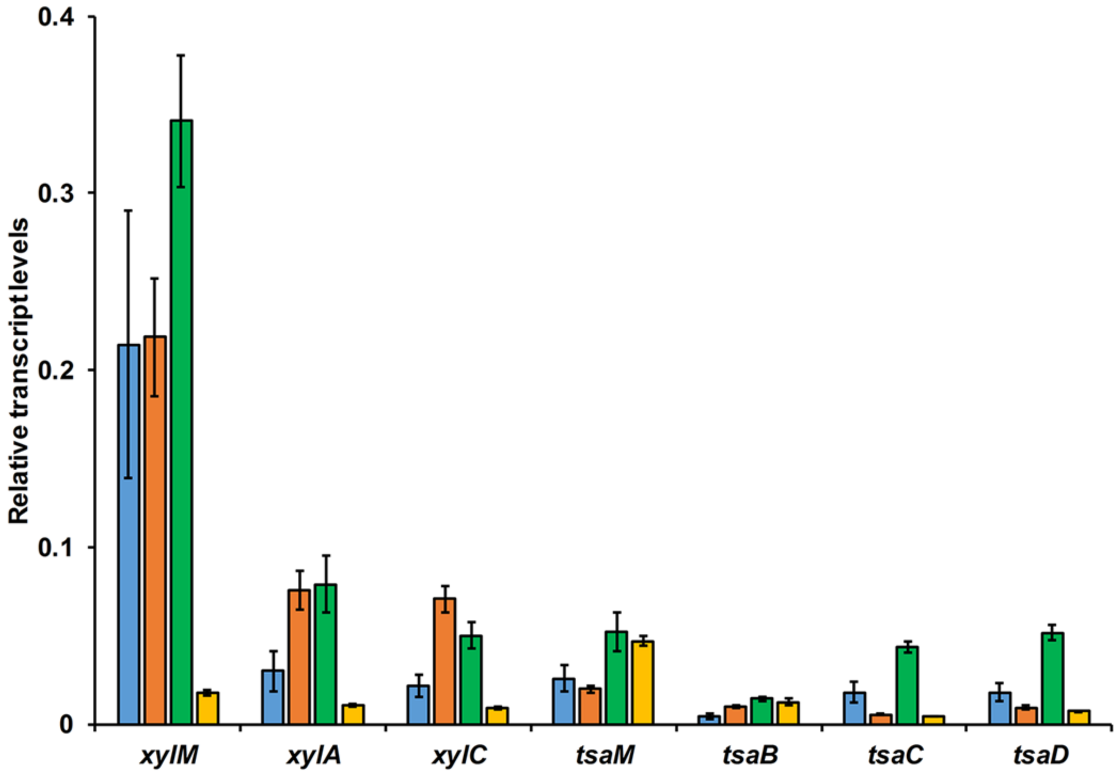


**Supplementary Figure 5. Relative transcript levels of *xylM*, *xylA*, *xylC*, *tsaM*, *tsaB*, *tsaC* and *tsaD* in *E. coli* DH5α cells harboring plasmids corresponding to Pi (blue box), Pii (orange box), Piii (green box) and Piv (yellow box), respectively, in the exponential phase.** Values and error bars represent the mean and s.d. of triplicate experiments.

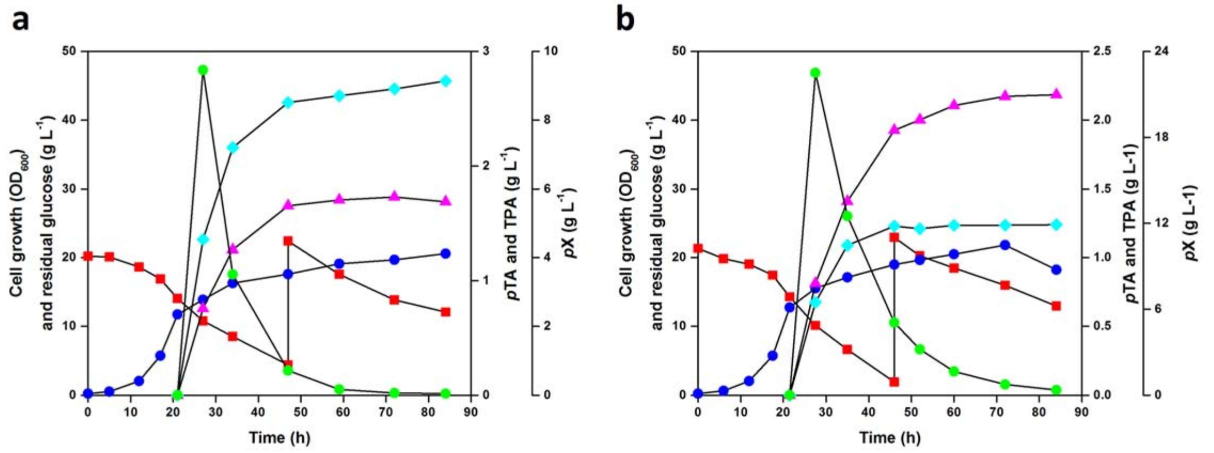


**Supplementary Figure 6. Preliminary two-phase partitioning fermentation profiles of *E. coli* DH5α harboring Piii for TPA production from 10 g (a) and 20 g (b) *p*X, respectively.** Symbols are: blue circle, cell growth ($OD_{600}$); red rectangle, residual glucose concentration (g $L^{-1}$); pink triangle, *p*TA concentration (g $L^{-1}$); light blue diamond, TPA concentration (g $L^{-1}$); green circle, residual *p*X concentration in oleyl alcohol (g $L^{-1}$).

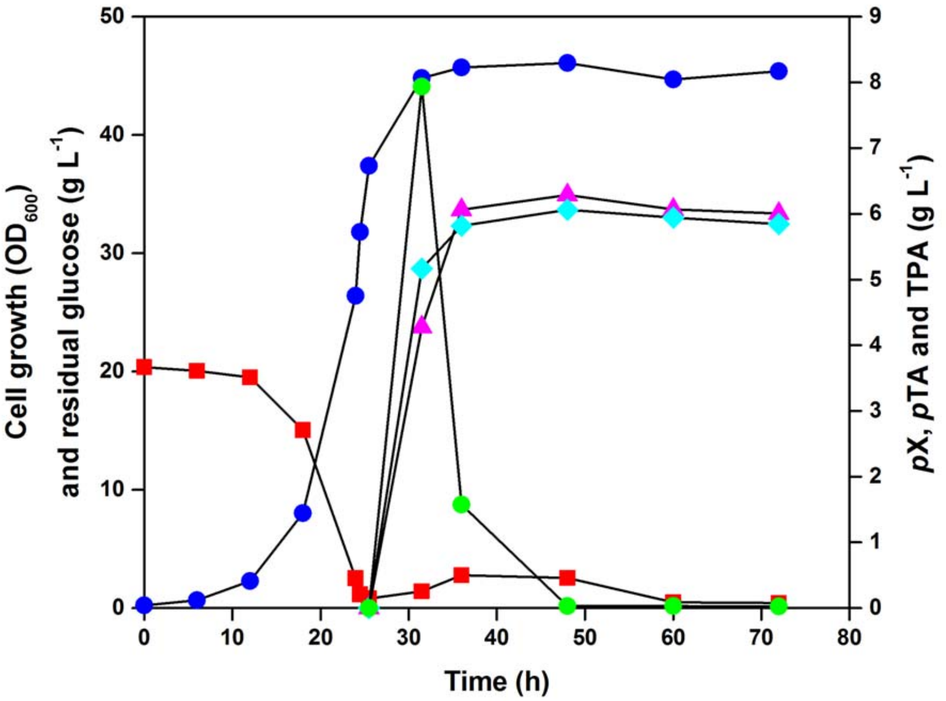


**Supplementary Figure 7. Two-phase partitioning DO-stat fed-batch fermentation profile of *E. coli* DH5α harboring Piii for TPA production from 20 g *p*X.** Symbols are: blue circle, cell growth ($OD_{600}$); red rectangle, residual glucose concentration (g $L^{-1}$); pink triangle, *p*TA concentration (g $L^{-1}$); light blue diamond, TPA concentration (g $L^{-1}$); green circle, residual *p*X concentration in oleyl alcohol (g $L^{-1}$).

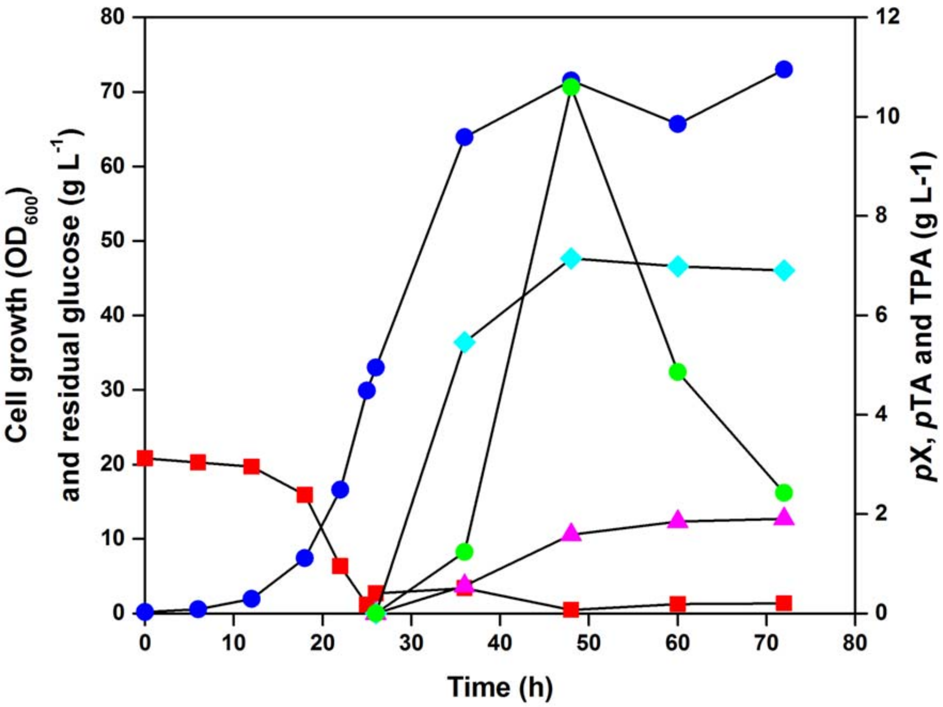


**Supplementary Figure 8. Two-phase partitioning fermentation profile of *E. coli* DH5α harboring Piii for TPA production from 20 g *p*X under DO-stat fed-batch conditions with reduced *p*X feeding rate.** Symbols are: blue circle, cell growth ($OD_{600}$); red rectangle, residual glucose concentration (g $L^{-1}$); pink triangle, *p*TA concentration (g $L^{-1}$); light blue diamond, TPA concentration (g $L^{-1}$); green circle, residual *p*X concentration in oleyl alcohol (g $L^{-1}$).

## SUPPLEMENTARY TABLES

**Supplementary Table 1. Various chemical processes for TPA manufacturing from *p*X.**

| Process | Commercialization | Reaction conditions | Characteristics | Reference |
|---|---|---|---|---|
| Amoco Process | Commercialized by BP | T: 175~225 $^oC$;<br>P: 15~30 bar;<br>C: Co/Mn/Br;<br>S: acetic acid<br>O: air | Major TPA technology; Improved water recycling and liquid-solid separation methods | 1 |
| INVISTA Process | Commercialized by DuPont | Same as Amoco Process conditions | Upgraded overall plant arrangement and solid-liquid separation techniques; Advanced residue recovery | 2 |
| Dow-Inca Process | Commercialized by Dow | Same as Amoco Process conditions | Novel high-pressure filtration and agitation system employed for enhanced mass transfer | 3 |
| Eastman Kodak Process | Commercialized by Eastman Kodak | T: 120~175 $^oC$;<br>P: 7.5~15 bar;<br>C: Co/acetaldehyde;<br>S: acetic acid<br>O: air | High purity up to 99%; With acetic acid co-produced; No requirement of special materials for reactor coating due to replacement of bromide | 4 |
| Toray Process | Commercialized by Toray | T: 110~140 $^oC$;<br>P: 30 bar<br>C: Co/paraldehyde;<br>S: acetic acid<br>O: air | High purity up to 99%; No requirement of special materials for reactor coating due to replacement of bromide | 5 |
| Teijin Process | Commercialized by Teijin | T: 100~130 $^oC$;<br>P: 10 bar<br>C: Co<br>S: acetic acid<br>O: air | High purity up to 99%; No requirement of special materials for reactor coating due to omitting of bromide | 6 |
| Garcia-Verdugo's process | Not commercialized | T: 380 $^oC$;<br>P: 220 bar;<br>C: $MnBr_2$;<br>S: water<br>O: $H_2O_2$ | Simultaneously oxidizing xylene isomers in a continuous mode in supercritical water; Considerable reduction in overall energy input; Simplified downstream purification | 7 |

**Supplementary Table 1 (continued). Various chemical processes for TPA manufacturing from *p*X.**

| Process | Commercialization | Reaction conditions | Characteristics | Reference |
|---|---|---|---|---|
| Tashiro's process | Not commercialized | T: 100~150 $^{o}C$; P: 20~30 bar; C: NHPI/Co/Mn; S: acetic acid O: air | Combined catalytic system with NHPI; Promoted oxidation leading to high product yield without any halogen ion | [8] |
| Li's process | Not commercialized | T: 200 $^{o}C$; P: 15 bar; C: Co/Mn/Br; S: acetic acid O: $O_2/CO_2$ | Spray reactor system employed; Dramatic reduction in byproduct formation compared to conventional stirred reactor system | [9] |

**Supplementary Table 2. Technologies for producing *p*X from bio-based resources.**

| Company | Process description | Commercialization status | Reference |
|---|---|---|---|
| Gevo | Biomass-derived isobutanol by yeast fermentation, followed by chemical conversion to *p*X | Pilot facility in operation | [10] |
| Anellotech | Catalytic fast pyrolysis of lignocellulosic biomass to BTX, tradenamed Bio-TCAT™ | Pilot facility in operation | [11] |
| Virent | Aqueous phase catalytic reforming of biomass to hydrocarbons including BTX, tradenamed BioForming™ | Pilot facility in operation | [12] |
| Ensyn | Fast pyrolysis of biomass to BTX, tradenamed Rapid Thermal Process™ | Pilot scale in operation | [13] |
| Micromidas | Dehydration of C6 carbohydrates to CMF, hydrogenolized to DMF, followed by [4+2] Diels-Alder cycloaddition with ethylene to produce *p*X | Pilot facility in operation | [14] |
| University of North Carolina at Chapel Hill | Bio-based ethylene conversion to *p*X | Not available | [15] |

BTX, benzene, toluene and xylene; CMF, 5-chloromethylfurfural; DMF, 2,5-dimethylfuran; FDCA, 2,5-furandicarboxylic acid.

**Supplementary Table 3. Earlier studies on microbial conversion of *p*X into TPA.**

| Strains | Origin of isolation | Bioavailability of *p*X | TPA titer | Reference |
|---|---|---|---|---|
| Isolate IR10 that likely belongs to *Burkholderia* | Industrial wastewater bioreactor that is used to treat a waste stream containing *p*X, *p*TA and TPA | Bioavailable | 89.0 mg $L^{-1}$ | [16, 17] |
| A mixture of *Stenotrophomonas maltophilia* and *Comamonas testosterone* | Soil | Bioavailable | 5 mg $L^{-1}$ | [18, 19] |

**Supplementary Table 4. Candidates for xylene monooxygenase (XMO), benzyl alcohol dehydrogenase (BADH) and benzaldehyde dehydrogenase (BZDH), respectively.**

| Name | Size (aa) | Organism | Identity (%) | Accession No. |
|---|---|---|---|---|
| XMO | | | | |
| XylM | 376 | *P. putida* F1 | 100 | ABQ79033 |
| | 369 | *P. putida* mt-2, pWW0 | 37.2 | CAC86827 |
| | 375 | *P. chlororaphis* | 81.6 | AAC27438 |
| | 367 | *Novosphingobium aromaticivorans*, pNL1 | 34.2 | ABP64090 |
| XylA | 349 | *P. putida* F1 | 100 | ABQ79032 |
| | 350 | *P. putida* mt-2 | 41.1 | CAC86826 |
| | 346 | *N. aromaticivorans*, pNL1 | 42.7 | ABP64091 |
| BADH | | | | |
| XylB | 377 | *P. putida* DOT-T1E | 100 | AFO51333 |
| | 366 | *P. putida* mt-2, pWW0 | 53.6 | CAC86825 |
| | 371 | *Acinetobacter pittii* PHEA-2 | 52.5 | ADY81899 |
| | 365 | *N. aromaticivorans*, pNL1 | 52.0 | ABP64142 |
| | 371 | *Acinetobacter calcoaceticus* | 50.4 | AAC32671 |
| BZDH | | | | |
| XylC | 492 | *P. putida* KT2440 | 100 | AAN67564 |
| | 487 | *P. putida* mt-2 | 31.0 | CAC86828 |
| | 501 | *N. aromaticivorans*, pNL1 | 40.6 | ABP64093 |
| | 484 | *A. calcoaceticus* | 32.6 | AAC32670 |

**Supplementary Table 5. Strains and plasmids used in this study.**

| Name | Relevant genotype | Reference |
|---|---|---|
| *E. coli Strain* | | |
| DH5α | F⁻ *endA1 glnV44 thi-1 recA1 relA1 gyrA96 deoR nupG purB20 φ*80d*lacZ*ΔM15 Δ(*lacZYA-argF*)U169, hsdR17($r_K^-$ $m_K^+$), λ⁻ | Invitrogen |
| *Plasmids* | | |
| pTrc99A | $Ap^R$, *trc* promoter, pBR322 origin, *lac*$I^q$, 4.2-kb | Lab stock |
| pTac15K | $Km^R$, *tac* promoter, p15A origin, 4.0-kb | Lab stock |
| pTrcMA | pTrc99A derivative containing *xylM*, *xylA* from *P. putida* F1 | This study |
| pTrcC | pTrc99A derivative containing *xylC* from *P. putida* KT2440 | This study |
| pTrcMAC | pTrcMA derivative containing *xylC* from *P. putida* KT2440 | This study |
| pTrcMB | pTrc99A derivative containing *tsaM*, *tsaB* from *C. testosteroni* T-2 | This study |
| pTacCD | pTac15K derivative containing *tsaC*, *tsaD* from *C. testosteroni* T-2 | This study |
| pTacMBCD | pTacCD derivative containing *trc-tsaMB*-terminator cassette | This study |
| pTrcMAC-MBCD | pTrcMAC derivative containing RBS-*tsaMB* and RBS-*tsaCD* cassettes | This study |
| pTacMAC | pTac15K derivative containing *xylMA-xylC* from pTrcMAC | This study |
| pTrcMBCD | pTrcMB derivative containing RBS-*tsaCD* | This study |

Abbreviations: Ap, ampicillin; Km, kanamycin; R, resistance.

**Supplementary Table 6. Oligonucleotides used in this study.**

| Primer name | Sequence 5'-3' |
|---|---|
| pTrc(xylMA)-f | CGGACGGCCGGAACTCCTAAGGAATTCGAGCTCGGTACCC |
| pTrc(xylMA)-r | TACTTGATGTATTCCCACATATGGTCTGTTTCCTGTGTGA |
| xylMA(pTrc)-f | TCACACAGGAAACAGACCATATGTGGGAATACATCAAGTACTAC |
| xylMA(pTrc)-r | GGGTACCGAGCTCGAATTCCTTAGGAGTTCCGGCCGTCCG |
| pTrc(xylC)-f | CGACCATCATCCGCATTTGAGGAATTCGAGCTCGGTACCC |
| pTrc(xylC)-r | TCACTGGCAAATACCGCCATATGGTCTGTTTCCTGTGTGA |
| xylC(pTrc)-f | TCACACAGGAAACAGACCATATGGCGGTATTTGCCAGTGA |
| xylC(pTrc)-r | GGGTACCGAGCTCGAATTCCTCAAATGCGGATGATGGTCG |
| xylC(pTrcMA)-f | GAACTCCTAAGGAATTCGAGCTCGGTACCCGGGATGGCGGTATTTGCCAG |
| xylC(pTrcMA)-r | CAGCCAAGCTTGCATGCCTGCAGGTCGACTCTAGAGTCAAATGCGGATGATGGTC |
| tsaMB-f | AGACAGGAATTCATGTTCATCCGCAATTGCTGG |
| tsaMB-r | AGACAGGGATCCTCAGATGTCCAGGACCAGC |
| tsaCD-f | AGACAGGAATTCATGAACCTGAACAAACAAGTGG |
| tsaCD-r | AGACAGCTGCAGTCAGGCCACGTAGTGCATGAAC |
| trctsaMB-f | GGTGATGCCGGCCACGATGCGTCCGGCGTAGAGTTGACAATTAATCATCC |
| trctsaMB-r | GAAGCATTGGTGCACCGTGCAGTCGATAAGCTCCGAAGAGTTTGTAGAAACG |
| RBStsaMB-f | CATCATCCGCATTTGACTCTAGAGTCGACCTGCATCACACAGGAAACAGAC |
| RBStsaMB-r | GAATTCTGTTTCCTGTGTGATCAGATGTCCAGGACCAGCC |
| RBStsaCD-f | GGCTGGTCCTGGACATCTGATCACACAGGAAACAGAATTC |
| RBStsaCD-r | GCCAAAACAGCCAAGCTTGCATGCCTGCATCAGGCCACGTAGTGCA |
| xylMAC(pTac)-f | TGAGCGGATAACAATTTCACACAGGAAACAGCCATATGTGGGAATAC |
| xylMAC(pTac)-r | ACTCTAGAGGATCCCCGGGTACCGAGCTCGTCAAATGCGGATGATG |
| RBStsaCD(pTrcMB)-f | GCGGCGGCCGGCTGGTCCTGGACATCTGAGTCACACAGGAAACAGAATTC |
| RBStsaCD(pTrcMB)-r | AGCTTGCATGCCTGCAGGTCGACTCTAGAGTCAGGCCACGTAGTGCATG |
| xylM(RT)-f | CCTGGTACAAGTCTTTGC |
| xylM(RT)-r | CAGCATGTCAAGGATGG |
| xylA(RT)-f | TTGCTGCTGATTTCTGCGAG |
| xylA(RT)-r | ACTCTTTCCAACGGTATGCC |
| xylC(RT)-f | ACCGTCTACACCAACGTCAAC |
| xylC(RT)-r | ACCGCCTCGTTTTCGTCCTTG |
| tsaM(RT)-f | CCAAGACCTGCATCAAGAGC |
| tsaM(RT)-r | CAGAAGTAATCGACGATGTC |
| tsaB(RT)-f | GGTGTGGATTCACGAAAAGC |

**Supplementary Table 6 (continued). Oligonucleotides used in this study.**

| Primer name | Sequence 5’-3’ |
|---|---|
| tsaB(RT)-r | ATGGCGTAGATGGGCGTGAC |
| tsaC(RT)-f | ATGGCGACCGAACTCAAC |
| tsaC(RT)-r | CGTTGTTCACCACGATGTG |
| tsaD(RT)-f | CATGGAGGTGTTCAAGTTC |
| tsaD(RT)-r | TTGAGCACGATGGAACAACC |

## SUPPLEMENTARY REFERENCES


1. Raghavendrachar P, Ramachandran S. Liquid-phase catalytic oxidation of *p*-xylene. *Ind. Eng. Chem. Res.* **31**, 453-462 (1992).
2. Turner JA, Royall DJ, Hugall DS, Jones GH, Woodcock DC. Production of terephthalic acid by liquid-phase oxidation. WO Patent 9,838,150 (1998).
3. Piras L, Schena S, Chiarelli M, Soro L. Process for the recovery of purified terephthalic acid (PTA). US Patent 6,639,104 (2001).
4. Kiefer JE, Phillips WV, Woodruff TE. Process for the purification of terephthalic acid. US Patent 4,605,763 (1986).
5. Nakaoka K, Miyama Y, Matsuhisa S, Wakamatsu S. Preparation of Terephthalic Acid Using Paraldehyde Promotor. *Ind. Eng. Chem. Prod. Res. Dev.* **12**, 150-155 (1973).
6. Ichikawa Y, Yamashita G, Tokashiki M, Yamaji T. New Oxidation Process for Production of Terephthalic Acid from p-Xylene. *Ind. Eng. Chem.* **62**, 38-42 (1970).
7. Garcia-Verdugo E, Fraga-Dubreuil J, Hamley PA, Thomas WB, Whiston K, Poliakoff M. Simultaneous continuous partial oxidation of mixed xylenes in supercritical water. *Green Chem.* **7**, 294-300 (2005).
8. Tashiro Y, Iwahama T, Sakaguchi S, Ishii Y. A New Strategy for the Preparation of Terephthalic Acid by the Aerobic Oxidation of *p*-Xylene using N-Hydroxyphthalimide as a Catalyst. *Adv. Synth. Catal.* **343**, 220-225 (2001).
9. Li M, Niu F, Zuo X, Metelski PD, Busch DH, Subramaniam B. A spray reactor concept for catalytic oxidation of *p*-xylene to produce high-purity terephthalic acid. *Chem. Eng. Sci.* **104**, 93-102 (2013).
10. Peters MW, Taylor JD, Jenni M, Manzer LE, Henton DE. Integrated Process to Selectively Convert Renewable Isobutanol to *p*-Xylene. US Patent 20,110,087,000 (2011).
11. Huber GW, Cheng YT, Carlson T, Vispute T, Jae J, Tompsett G. Catalytic pyrolysis of solid biomass and related biofuels, aromatic, and olefin compounds. US Patent 8,277,643 (2012).
12. Cortright RD, Blommel PG. Synthesis of liquid fuels and chemicals from oxygenated hydrocarbons. US Patent 8,053,615 (2011).
13. Freel B. Rapid thermal conversion of biomass. US Patent 7,905,990 (2011).
14. MASUNO MN, *et al.* Methods of producing para-xylene and terephthalic acid. US Patent 9,260,359 (2016).
15. Lyons TW, Guironnet D, Findlater M, Brookhart M. Synthesis of *p*-Xylene from Ethylene. *J. Am. Chem. Soc.* **134**, 15708-15711 (2012).
16. Bramucci MG, *et al.* Pure bacterial isolates that convert p-xylene to terephthalic acid. *Appl. Microbiol. Biotechnol.* **58**, 255-259 (2002).
17. Bramucci MG, McCutchen CM, Nagarajan V, Thomas SM. Terephthalic acid producing Proteobacteria. US Patent 6,461,840 (2002).
18. Wang J, Tian J, Xu J, Gao P. Elementary studies on biotransformation of *p*-xylene into terephthalic acid. *Wei Sheng Wu Xue Tong Bao* **33**, 17-21 (2006).
19. Sang P, Tian J, Gao P, Xu GW. Studies on Pathway of Producing Terephthalic Acid by Microorganism Coordinated Catalysis. *Chin. J. Anal. Chem.* **36**, 1024-1028 (2008).
